# UHF RFID Tags Installation Methods for Vehicle Navigation on Paved and Unpaved Roads

**DOI:** 10.3390/s26154794

**Published:** 2026-07-28

**Authors:** Gabriela Maria Castro Gonzalez, Takayuki Kawaguchi, Dai Nakamura, Kenji Kurokawa, Takeshi Kawamura

**Affiliations:** 1 Co-Creative Engineering, Doctoral Program, Graduate School of Engineering, Kitami Institute of Technology, Kitami 090-8507, Hokkaido, Japan; d3247180014@std.kitami-it.ac.jp; 2 Social Environment Engineering, Kitami Institute of Technology, Kitami 090-8507, Hokkaido, Japan; kawa@mail.kitami-it.ac.jp (T.K.); dnaka@mail.kitami-it.ac.jp (D.N.); 3 Information and Communications Engineering, Kitami Institute of Technology, Kitami 090-8507, Hokkaido, Japan; kurokawa@mail.kitami-it.ac.jp

**Keywords:** UHF RFID, communication range, unpaved roads, concrete blocks

## Abstract

This study investigated the communication performance of ultrahigh-frequency radio-frequency identification (RFID) systems with embedded RFID tags on challenging roads. Surface-mounted RFID tags are used for vehicle guidance. However, in snowy regions, they are vulnerable to damage caused by snow removal. Accordingly, RFID tags were embedded beneath the road surface, and the influence of an installation angle of 30° on communication performance was evaluated. Using the appropriate installation angle for asphalt embedding, this study investigated unpaved roads, where tag stability is influenced by water flow and soil displacement. To improve durability, RFID tags were attached to geocell-based reinforcement structures using polyvinyl chloride (PVC) pipe holders. The communication performance was comparable to that of asphalt-embedded configurations. To further improve installation, a concrete-block-based embedding approach was investigated. Gravel concrete and mortar concrete block produce significant electromagnetic attenuation, which reduces the communication range. Therefore, a hybrid design using PVC pipes embedded in commercially available concrete blocks and holes filled with crushed stone was developed. This design achieves the communication range required for reliable vehicle navigation while simplifying installation and enabling application on natural soil unpaved roads without geocell reinforcement.

## 1. Introduction

Vehicle navigation under adverse environmental conditions remains a significant challenge for autonomous and assisted driving systems. In snowy and mountainous regions, such as Hokkaido, Japan, heavy snowfall, whiteout conditions, and complex terrain reduce the reliability of conventional localization technologies. Optical sensors, including cameras and light detection and ranging (LiDAR), experience degraded performance because of snow flakes and reduced visibility, whereas global navigation satellite system (GNSS)-based positioning may suffer from signal blockage and multipath effects caused by surrounding terrain and infrastructure [1,2,3,4,5]. Consequently, infrastructure-assisted localization technologies have attracted growing attention as complementary positioning methods that maintain reliable vehicle guidance under severe environmental conditions.

Among these technologies, electromagnetic guidance and magnetic marker systems have demonstrated high localization accuracy on paved roads [6,7,8,9]. However, these approaches require dedicated infrastructure and have not been widely investigated for unpaved road environments.

Previous studies established the feasibility of Radio-frequency identification (RFID)-based vehicle navigation by embedding passive ultrahigh-frequency (UHF) RFID tags beneath paved roads and detecting them using antennas mounted on the vehicle [5]. Subsequent investigations demonstrated that the communication performance is strongly influenced by the burial angle of the RFID tag and showed that an installation angle of approximately 30° provides the communication range required for reliable vehicle localization [10]. Based on these findings, a four-antenna RFID vehicle guidance system capable of estimating a vehicle’s lateral position within the driving lane was developed and successfully validated under heavy snowfall conditions, demonstrating stable vehicle guidance even when conventional localization methods became unreliable [11].

Although RFID-based vehicle navigation has shown promising performance on paved roads, extending the technology to unpaved roads introduces additional challenges. Unlike asphalt pavements, unpaved roads are susceptible to deformation caused by rainfall, flowing water, freeze–thaw cycles, and repeated vehicle loading, making it difficult to maintain the position and orientation of buried RFID tags. To address this issue, our previous study proposed a polyvinyl chloride (PVC) holder attached to a geocell reinforcement structure to maintain the RFID tag at the required 30° installation angle [12]. Experimental results demonstrated communication performance comparable to that achieved on paved roads and confirmed the feasibility of RFID-based vehicle guidance on reinforced unpaved roads. However, the installation process requires dedicated geocell reinforcement, careful alignment of the PVC holder, and fixation using vinyl ties, resulting in increased construction complexity and reduced installation efficiency.

We considered embedding RFID tags in concrete blocks as a simple way to secure them to geocells. The objective of this study is to ascertain the type of concrete block that would provide the adequate communication range for the RFID system. Nevertheless, the electromagnetic properties of concrete can significantly influence the communication performance of passive RFID systems. Embedding RFID tags within concrete has also been investigated in applications unrelated to vehicle navigation. Laheurte et al. [13] developed an embedded UHF RFID system for monitoring the durability of concrete structures and demonstrated that wireless communication could be maintained through concrete materials. However, their work focused on structural health monitoring rather than vehicle navigation and employed battery-assisted RFID tags instead of passive RFID technology.

Since no previous studies have evaluated the communication range of passive UHF RFID tags embedded in concrete blocks, this study experimentally measures and compares the communication performance of mortar concrete blocks, gravel concrete blocks, full-sized commercial concrete blocks, and half-sized commercial concrete blocks with that of previously developed installation methods. Communication performance is evaluated through communication range measurements and geometrical analyses, followed by vehicle guidance experiments on snow-covered unpaved roads. The experimental results demonstrate that the half-sized commercial concrete block can be integrated into geocells without requiring additional installation steps while still providing the communication range necessary for reliable vehicle guidance. RFID tags can be embedded into natural unpaved road without geocell, when using commercially available concrete blocks. Furthermore, all analyses and design recommendations presented in this study are derived from experimental validation rather than theoretical.

## 2. Related Work

### 2.1. Vehicle Localization Technologies

Reliable vehicle localization is one of the fundamental requirements for autonomous driving. Conventional localization systems primarily rely on global navigation satellite systems (GNSS), light detection and ranging (LiDAR), cameras, radar, and inertial navigation systems (INS). GNSS provides absolute positioning over large areas but its accuracy deteriorates in environments affected by signal obstruction and multipath effects, such as mountainous regions and urban canyons [1,6,7,8,9,14,15,16,17,18,19,20,21,22,23,24]. LiDAR- and vision-based localization methods, including simultaneous localization and mapping (SLAM), achieve high positioning accuracy under favorable environmental conditions [3,4]. However, these approaches are sensitive to adverse weather conditions, including heavy snowfall, fog, and poor illumination, which reduce feature visibility and sensor reliability [2,3,4,14].

Infrastructure-assisted localization methods have therefore attracted considerable attention. Electromagnetic guidance systems can estimate vehicle lateral displacement with high precision, while magnetic marker systems have demonstrated reliable localization for automated buses operating on predefined routes [6,7,8,9]. Although these technologies provide accurate localization, they require dedicated infrastructure and have been primarily developed for paved roads.

### 2.2. RFID-Based Vehicle Navigation

Passive UHF-RFID has been widely investigated as an infrastructure-assisted localization technology because RFID tags can provide absolute position information independent of satellite reception or environmental visibility. Previous studies have proposed RFID-based localization using received signal strength indicator (RSSI), phase measurements, multilateration, and dense RFID tag deployment for both indoor and outdoor positioning applications [15,16,17,18,19,20,21,22,23,24]. These approaches have demonstrated improved positioning accuracy; however, many require sophisticated signal processing, dense tag deployment, or extensive computational resources.

## 3. System Overview

**Figure 1:**
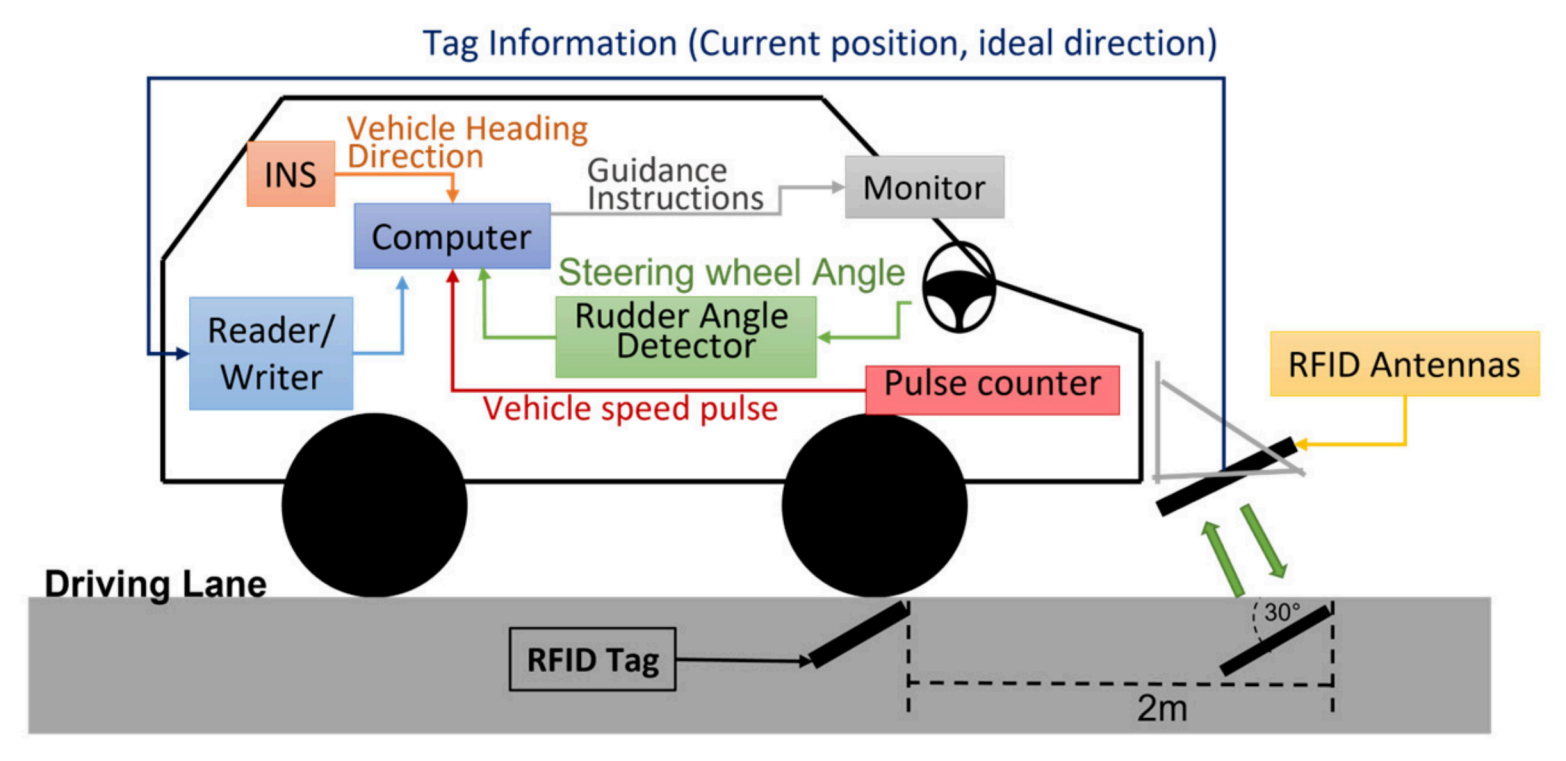
Sideview of the vehicle guidance system [12].

The vehicle guidance system developed in previous studies utilizes passive UHF-RFID technology to provide lane-level vehicle localization and navigation. The system consists of three main components: passive RFID tags embedded beneath the roadway, multiple RFID antennas mounted on the vehicle, and a laptop that estimates the vehicle position and generates real-time driving guidance. By combining RFID detection data with heading information obtained from an inertial navigation system (INS), the system continuously determines the vehicle position relative to the center of the driving lane. A schematic overview of the system is presented in Figure 1 [5,12].

Passive RFID tags containing lane identification information are embedded beneath the center of the driving lane at intervals of 2.0 m. Each tag is installed at an angle of approximately 30° with respect to the road surface to obtain an adequate communication with the vehicle-mounted antennas, as established in previous studies [10]. Four UHF RFID antennas are installed laterally at the front of the vehicle with a spacing of 0.40 m between adjacent antennas. The antennas are inclined to match the orientation of the buried RFID tags, thereby improving communication reliability. As the vehicle travels over an RFID tag, the RFID reader records the detection status of each antenna. Because the four antennas observe different portions of the communication region, the combination of detected signals varies according to the lateral position of the vehicle within the lane. The laptop integrates the RFID detection results with the vehicle heading measured by the INS to estimate the vehicle position in real time. Based on these detection combinations, the driving lane is divided into seven lateral localization regions, enabling continuous vehicle guidance [11].

The communication range between the RFID antennas and buried tags is one of the most important parameters affecting system performance. If the communication range is too narrow, tags may not be detected continuously during vehicle operation, resulting in discontinuous localization. Conversely, an excessively large communication range increases the overlap between adjacent detection regions, reducing the accuracy of lateral position estimation. Therefore, maintaining an appropriate communication range is essential for achieving both reliable tag detection and accurate vehicle localization.

Previous investigations demonstrated that an effective lateral communication range of approximately 0.85 m provides an appropriate balance between detection continuity and positioning accuracy for the four-antenna guidance system [5,10]. Consequently, the installation geometry of both the RFID tags and antennas was designed to achieve this communication range.

### Stick Type RFID Tags, Reader/Writer, and Antennas

The RFID tag used throughout this study is the G2-Stick 1Holl M4QT passive UHF RFID tag (Sato Material, formerly Nitta Industries Co., Kobe, Ltd., Japan). The tag complies with the EPC global Class 1 Generation 2 (EPC C1 Gen2) air interface protocol (ISO/IEC 18000-63 [25]) and operates within the UHF frequency band of 860–960 MHz. It has a cylindrical stick-type housing measuring ${1.15\;\times 10}^{-1}$ m in length and ${0.8\;\times 10}^{-3}$ m in diameter and provides IP67-equivalent protection against dust and water ingress, making it suitable for long-term underground installation. The housing encloses a Dogbone Monza 4QT UHF RFID inlay (Impinj, Seattle, WA, USA), which consists of a flexible RFID inlay wrapped around a plastic rod to form the cylindrical stick configuration.

**Figure 2:**
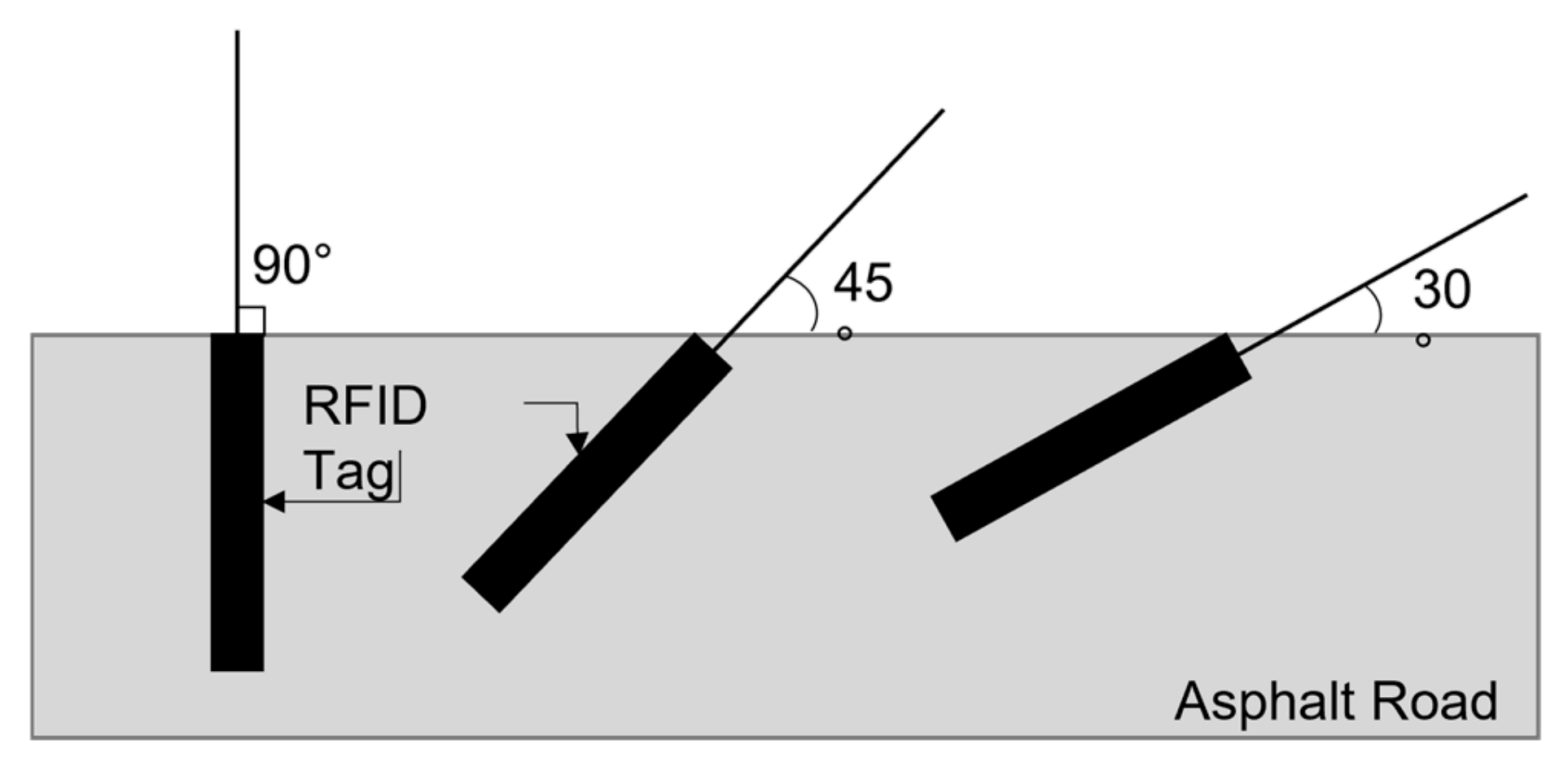
Side view of buried RFID tags with the angles of 90°, 45°, and 30° [10].

Passive UHF stick-type RFID tags exhibit directional radiation characteristics, with electromagnetic waves primarily propagating perpendicular to the longitudinal axis of the tag (Figure 2) [10]. Consequently, the installation angle of the buried RFID tag significantly influences the communication performance with the vehicle-mounted antennas. Earlier investigations evaluated installation angles of 90°, 45°, and 30° relative to the road surface and demonstrated that tilting the RFID tag substantially improves electromagnetic coupling by directing the strongest radiation pattern toward the antenna.

When the RFID tag was installed vertically (90°), the asphalt pavement attenuated the electromagnetic waves, limiting the effective communication height to approximately 0.09 m. Tilting the tag to 45° increased the communication height to approximately 0.27 m, while an installation angle of 30° produced a communication range exceeding 0.60 m, satisfying the requirements for continuous vehicle detection [10]. Accordingly, both the RFID tags and the vehicle-mounted antennas were installed at an angle of 30° to maximize communication efficiency.

RFID communication was performed using four UHF RFID antennas (A5010, Circular polarized, Times-7, Petone, Lower, New Zealand) mounted at the front of the experimental vehicle. The antennas were arranged laterally with a spacing of 0.40 m and installed at the same 30° angle as the buried RFID tags to maintain parallel alignment between the reader antennas and tag antennas. The four antennas were connected to an Impinj Speedway R420 RFID reader/writer (Impinj Inc., Seattle, WA, USA), which complies with the EPCglobal Class 1 Generation 2 (EPC C1 Gen2) protocol and operates within the Japanese UHF RFID frequency band of 916.8–920.4 MHz.

Figure 2 illustrates the installation configurations investigated in the previous study. Under the selected 30° configuration, the communication region between the buried RFID tag and the vehicle-mounted antenna formed an approximately elliptical detection area. Experimental evaluations demonstrated an effective lateral communication range of approximately 0.75–0.85 m for an antenna mounted at a height of 0.40 m above the road surface [5,10]. This communication range provides a suitable balance between detection continuity and lateral positioning accuracy and was therefore adopted as the design criterion for the vehicle guidance system.

**Figure 3:**
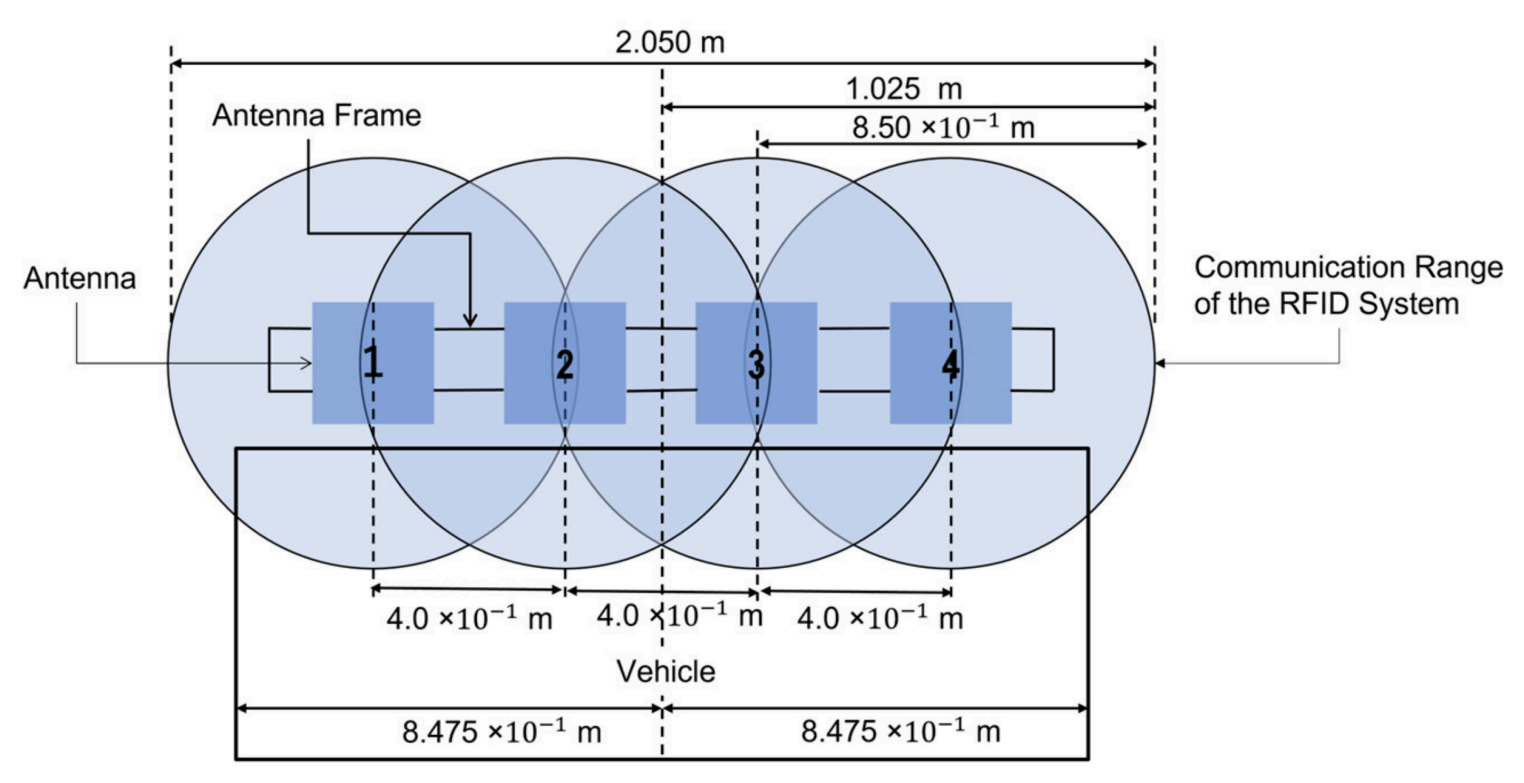
RFID antenna frame measurements and communication range of the system with a diameter of ${8.50\;\times 10}^{-1}$ m [11].

To improve localization accuracy while reducing the number of buried RFID tags, a four-antenna configuration was subsequently developed [11]. As illustrated in Figure 3, the overlapping communication regions of the four antennas enable a single RFID tag to be detected by multiple antennas simultaneously. The resulting antenna detection combinations divide the driving lane into seven lateral localization regions, allowing the laptop to estimate the vehicle position.

The communication characteristics established on paved roads form the foundation of the present study. By preserving the same RFID hardware, antenna geometry, installation angle, communication range, and operating parameters, the performance of the proposed embedding methods can be evaluated independently, ensuring that any observed differences arise from the embedding structures rather than the RFID guidance system itself. The effect of snow cover on RFID communication was examined by measuring the communication range under both snow-free and snow-covered conditions using the same experimental setup, which consisted of a plywood board with gridlines marked on its surface. For the snow-covered condition, the gridded plywood board was placed on a 0.2 m-high wooden frame filled with snow at the test site, while the reader antenna was maintained at a constant height of 0.4 m above the snow surface. The measured communication range remained essentially the same in the presence of the snow layer.

These findings are particularly relevant to Hokkaido, Japan, where winter roads are commonly covered by approximately 0.2 m of compacted snow and ice. Vehicle guidance experiments under these conditions demonstrated localization accuracy comparable to that obtained on snow-free pavement, and no degradation in guidance performance was observed after the snow melted in spring. These results indicate that seasonal snow accumulation has little influence on the communication performance of the proposed RFID-based vehicle guidance system.

## 4. Challenges of Maintaining RFID Communication on Unpaved Roads

Although the RFID-based vehicle guidance system has demonstrated reliable localization performance on paved roads, extending the technology to unpaved roads presents additional challenges. Unlike asphalt pavements, unpaved road surfaces are continuously affected by rainfall, flowing water, repeated vehicle loading, and material settlement. These environmental factors cause displacement of the roadbed material, which can alter the position and orientation of buried RFID tags. Because the communication performance of passive UHF RFID tags is highly dependent on their installation geometry, even small changes in tag orientation may reduce the communication range and degrade vehicle localization accuracy.

To improve the mechanical stability of unpaved roads, geocell reinforcement systems have been widely adopted. Geocells confine granular filler materials within interconnected cells, thereby reducing soil displacement and improving the structural stability of the roadbed.

In order to install the RFID tag on a geocell structure, a polyvinyl chloride (PVC) holder was previously developed that fixes the RFID tag at an installation angle of 30° and attaches it directly to the geocell structure using vinyl ties [12].

### 4.1. PVC Holder [12]

**Figure 4:**
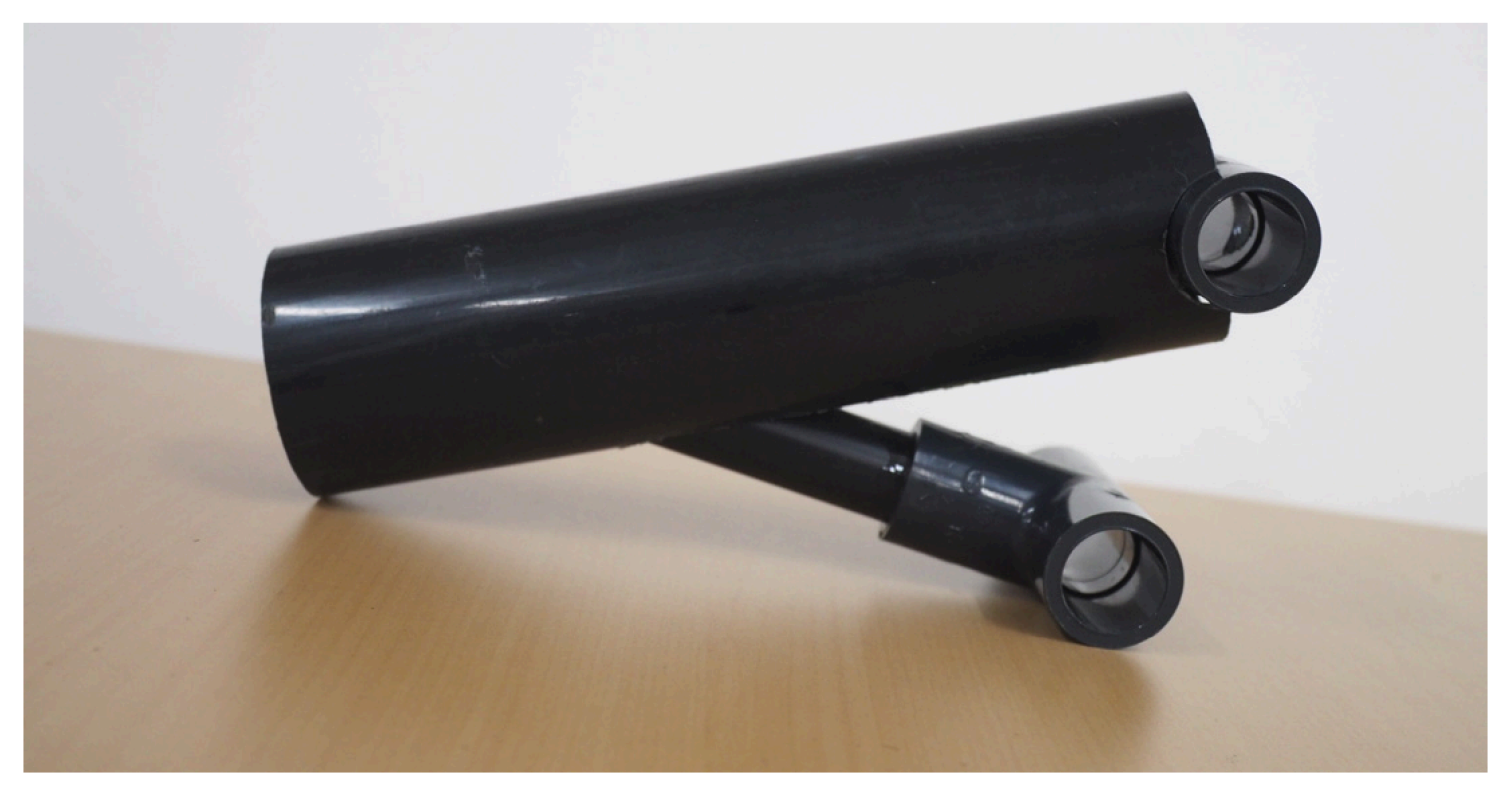
Tag holder with cover.

An RFID tag holder developed in our previous study and fabricated in our laboratory (Figure 4) was used to maintain the orientation of the passive UHF RFID tags under unpaved road conditions [12]. The holder was fabricated from a PVC pipe with an outer diameter of ${1.8\times 10}^{-2}$ m and an inner diameter of ${1.3\times 10}^{-2}$ m. It was designed to position the RFID tag at the required 30° installation angle. The RFID tag was enclosed within the holder to protect it from environmental exposure while maintaining a stable orientation. A detailed description of the holder installation procedure is provided in our previous study [12].

However, because the holder is attached to the geocell using vinyl ties, its installation depends on geocell reinforcement, increasing construction complexity and limiting its applicability to other types of unpaved road.

The experimental driving lane used in this study consisted of a 50 m unpaved test section reinforced with geocells and filled with C-40 crushed stone having particle sizes ranging from 0 m to ${4.0\times 10}^{-2}$ m. Prior to the driving test, a PVC holder and an RFID tag were attached to a geocell filled with C-40, and the communication range was measured in advance. Building upon the communication characteristics established in previous studies, this research investigates several concrete-based RFID embedding structures as alternatives to the conventional PVC holder. The objective is to simplify the installation process while maintaining the communication performance and structural stability required for reliable RFID-based vehicle guidance on unpaved roads.

### 4.2. Mortar Concrete Block

**Figure 5:**
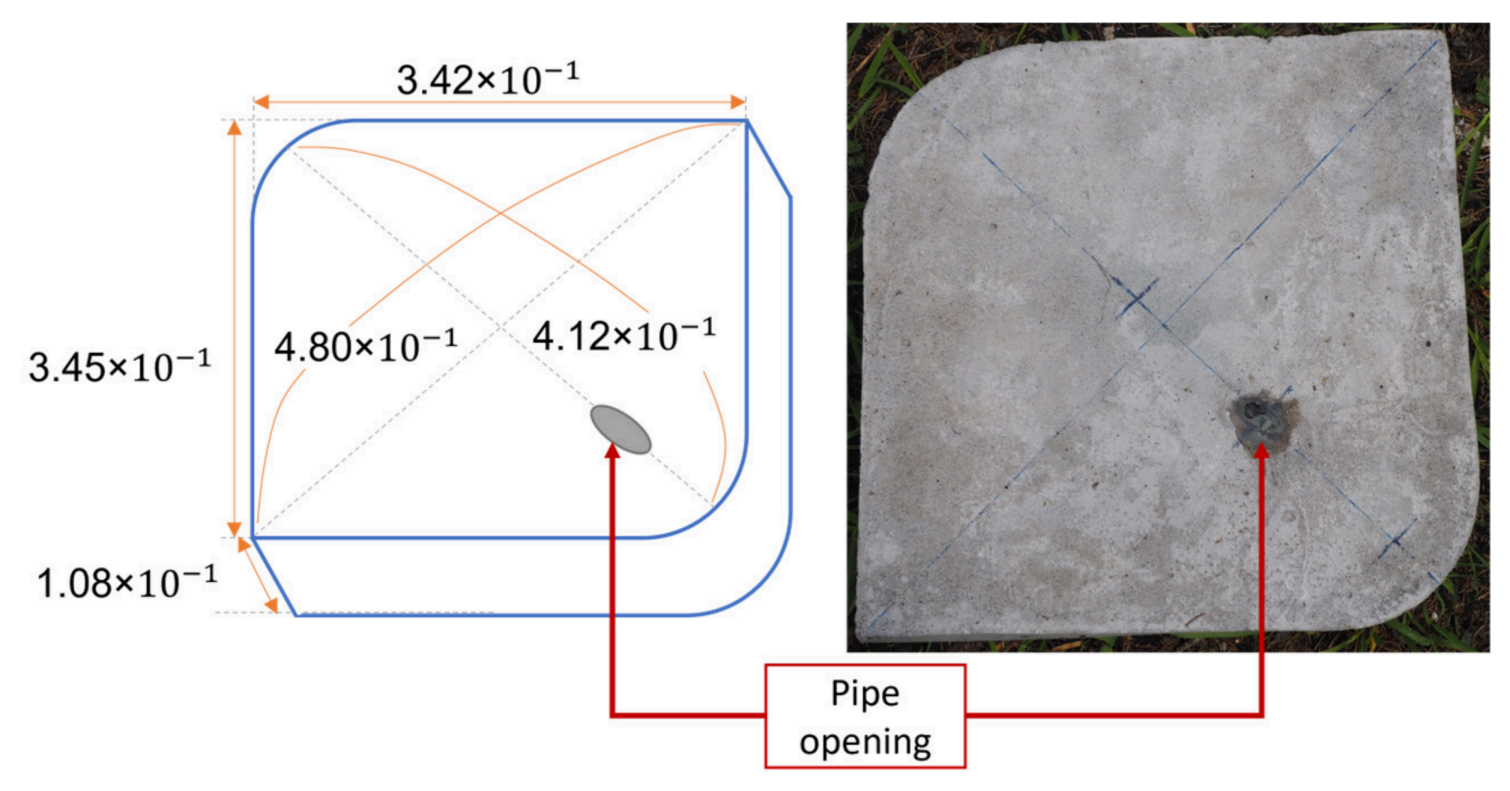
Measurements of the mortar concrete block.

To reduce effort on the installation procedure, a mortar concrete block was developed as an integrated RFID embedding structure (Figure 5). Unlike the PVC holder, the proposed block does not require a dedicated holder assembly and can be directly embedded into the road. The mortar concrete block was fabricated from a mixture of cement, sand, and water, and molded to fit the geocell structure. The block had an approximately square shape, with the dimensions of ${3.42\times 10}^{-1}$ m in width, ${3.45\times 10}^{-1}$ m in depth, and ${1.0\times 10}^{-1}$ m in height. A PVC pipe with the same dimensions as the PVC holder (${1.8\times 10}^{-2}$ m outer diameter and ${1.3\times 10}^{-2}$ m inner diameter) was embedded during block fabrication and trimmed to match the block surface. A stick-type RFID tag was then inserted into the pipe, which was sealed with silicone sealant to secure the tag and prevent the ingress of dust, moisture, and other contaminants.

This configuration was designed to maintain the required RFID tag orientation while reducing installation complexity and improving the structural integration of the embedding structure with the surrounding roadbed.

### 4.3. Gravel Concrete Block

**Figure 6:**
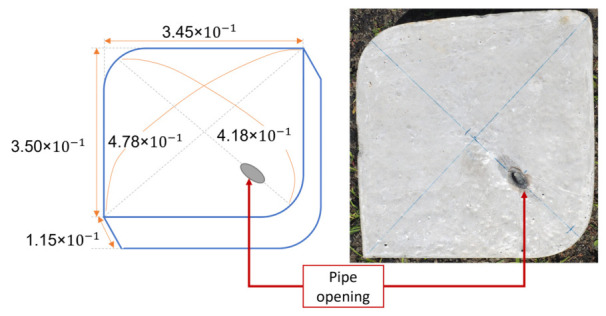
Measurements of the gravel concrete block with gravel.

A gravel concrete block was developed to investigate the influence of the embedding material on RFID communication performance (Figure 6). Compared with the mortar concrete block, the incorporation of coarse aggregates creates additional internal air voids, which are expected to improve electromagnetic wave propagation while maintaining adequate structural strength. The block was fabricated with dimensions of ${3.45\times 10}^{-1}$ m in width, ${3.50\times 10}^{-1}$ m in depth, and ${1.15\times 10}^{-1}$ m in height. As with the mortar concrete block, a PVC pipe was embedded at an installation angle of 30° to ensure a consistent RFID tag orientation throughout the experiments. As with the mortar concrete block, a PVC pipe identical to that described in Section 4.2 was embedded at a 30° installation angle. A stick-type RFID tag was inserted into the pipe and sealed with silicone sealant to protect it from dust, moisture, and other contaminants. The primary objective of this design was to evaluate whether modifying the block composition could improve communication performance while preserving the simplified installation procedure provided by the integrated embedding structure.

### 4.4. Commercially Available Concrete Blocks

**Figure 7:**
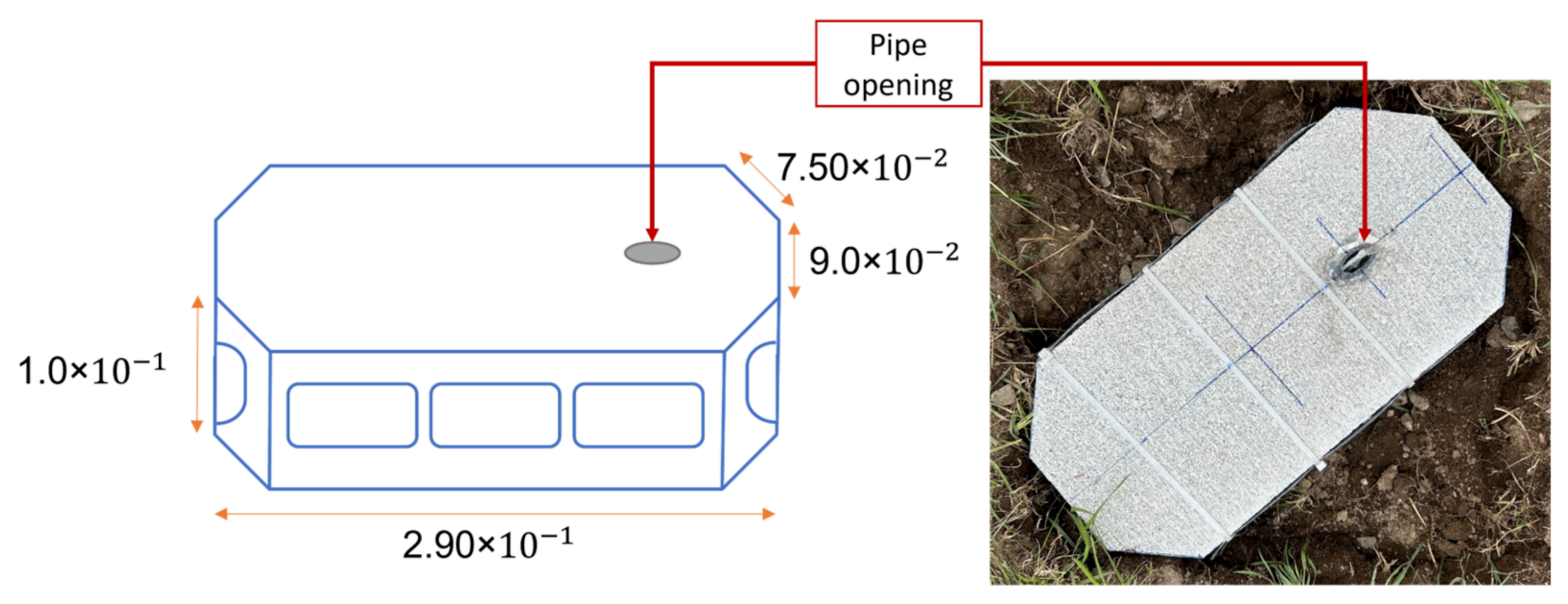
Measurements of the full-sized concrete block.

**Figure 8:**
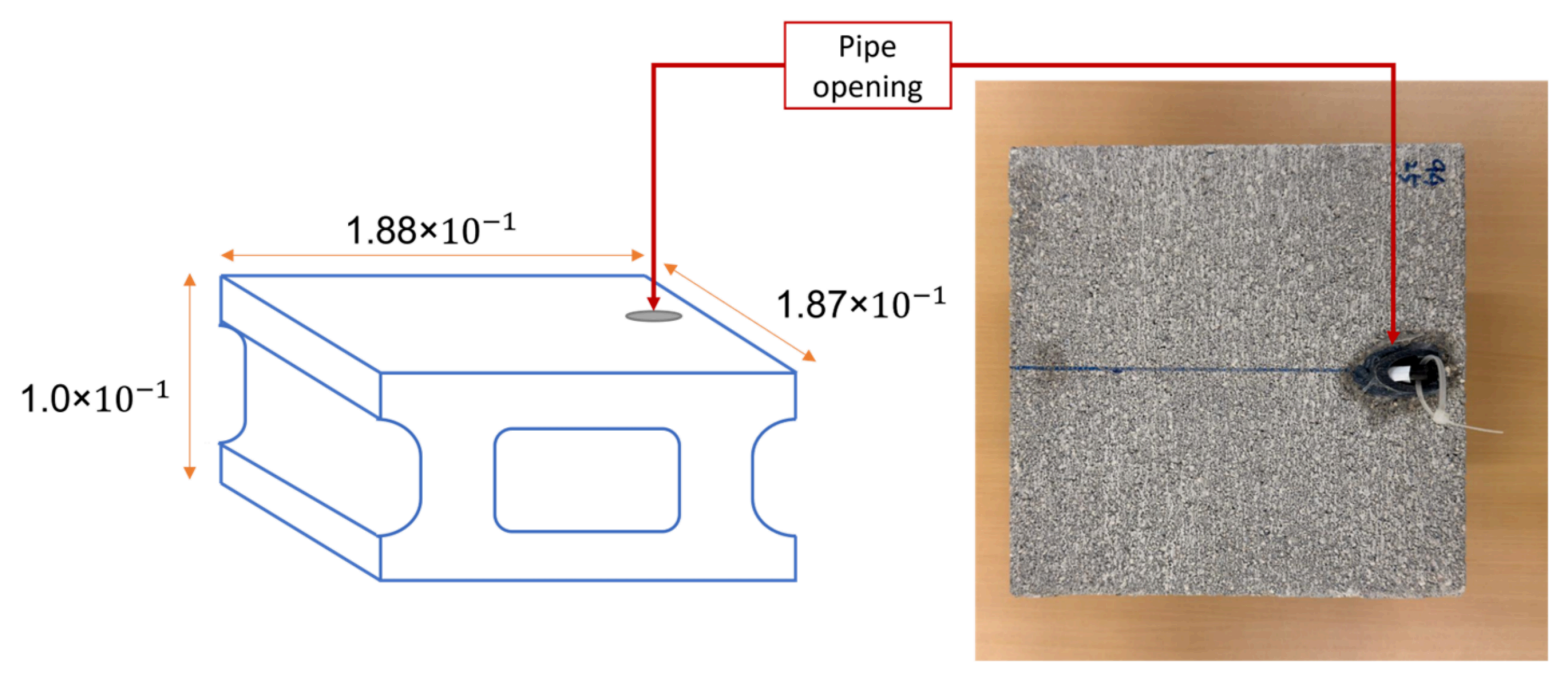
Measurements of the half-sized concrete block.

Commercially available concrete blocks compliant with the Japanese Industrial Standards (JIS) were also investigated as practical RFID embedding structures. In contrast to the custom-fabricated mortar and gravel concrete blocks, these products require minimal fabrication and therefore have the potential to further simplify field installation. Two configurations were evaluated: a full-sized concrete block (Figure 7) and a half-sized concrete block (Figure 8).

The full-sized block had the dimensions of ${2.90\times 10}^{-1}$ m in width, ${1.87\times 10}^{-1}$ m in depth, and ${1.0\times 10}^{-1}$ m in height. The block edges were trimmed to fit within the geocell structure, and a PVC pipe containing the RFID tag was embedded at a 30°. installation angle. The hollow cavities were filled with No. 5 crushed stone, with particle sizes ranging from ${1.30\times 10}^{-2}$ m to ${2.0\times 10}^{-2}$ m, and covered with geocell material to prevent aggregate loss during installation and operation.

A half-sized concrete block, with dimensions of ${1.88\times 10}^{-1}$ m in width, ${1.87\times 10}^{-1}$ m in depth, and ${1.0\times 10}^{-1}$ m in height, was developed to reduce the required installation area while maintaining the same RFID installation geometry. As with the full-sized block, the RFID tag was installed inside a PVC pipe positioned at 30°, and the cavity was filled with No. 5 crushed stone and covered with geocell material.

For both block configurations, the PVC pipe has the same dimensions as the PVC holder (${1.8\times 10}^{-2}$ m outer diameter and ${1.3\times 10}^{-2}$ m inner diameter). A 30° hole is drilled into the concrete block, the PVC pipe is inserted and secured at the bottom with silicone sealant, and the pipe is trimmed to match the block surface. A stick-type RFID tag is then inserted into the pipe, and the opening is sealed with silicone sealant to secure the RFID tag in place and prevent the ingress of dust, moisture, and other contaminants. Compared with the PVC holder, both concrete block configurations eliminate the need for a dedicated holder assembly while providing structural support for the embedded RFID tag. In particular, the half-sized block offers a more compact installation method that has the potential to maintain reliable communication performance while reducing construction complexity. Because the concrete block itself provides mechanical stability, this configuration may also be applicable to unpaved roads where geocell reinforcement is limited or unavailable.

## 5. Results of Communication Range Measurement

**Figure 9:**
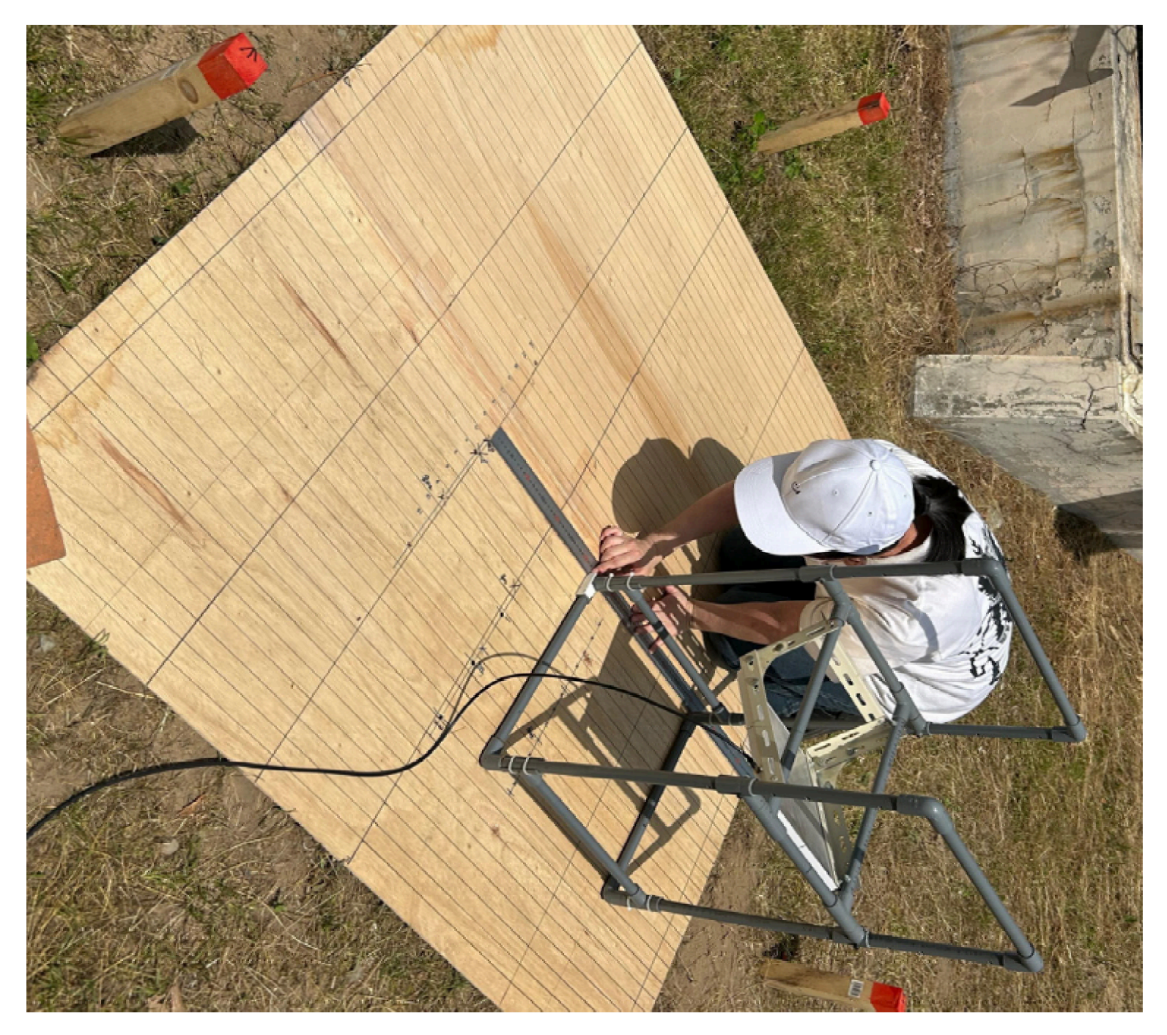
Measurement of the communication range with the antenna and plywood method.

The communication range was experimentally evaluated for four block types: mortar concrete, gravel concrete, full-sized commercial concrete, and half-sized commercial concrete. The communication range was measured using a plywood-based coordinate grid positioned above the embedded RFID tag, as shown in Figure 9. The Y-axis was aligned in the longitudinal direction of the RFID tag, whereas the X-axis was perpendicular to the tag axis. The measurement intervals were set to ${3.0\;\times \;10}^{-2}$ m along the Y-axis and ${5.0\;\times \;10}^{-2}$ m along the X-axis. During the measurements, the antenna frame was gradually moved toward the RFID tag position from the outer boundary of the measurement grid. The coordinates at which successful tag detection first occurred were recorded as communication boundaries. The measurements were repeated four to five times at each position to ensure repeatability and reduce experimental error. This procedure was conducted symmetrically on both sides of the tag axis to obtain the complete communication range.

**Figure 10:**
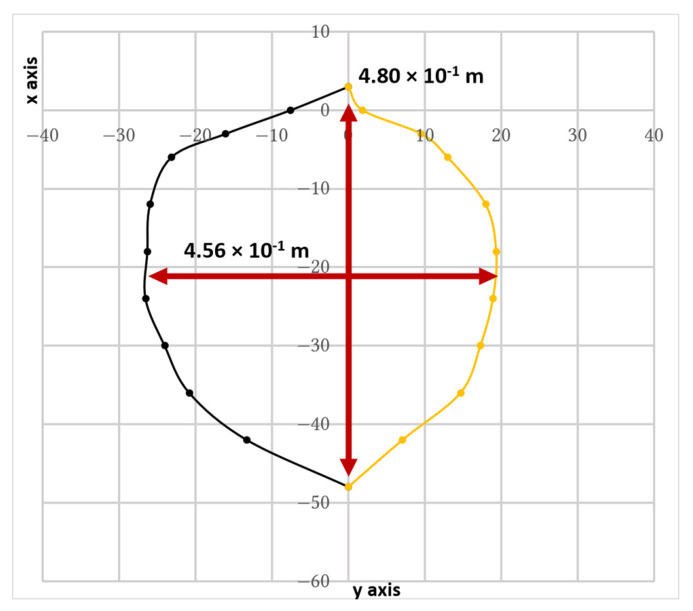
Communication range for the mortar concrete block.

**Figure 11:**
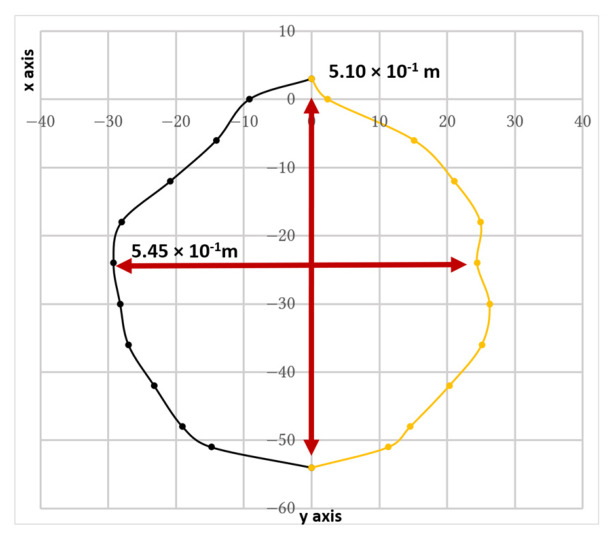
Communication range for the gravel concrete block.

For the mortar and gravel concrete blocks, the RFID reader was operated at a maximum antenna output power of 30 dBm. Both block types exhibited significant electromagnetic attenuation owing to their dense cement-based materials, which resulted in relatively limited communication ranges. Consequently, the maximum available antenna output power was required to obtain measurable communication performance. Despite operating at maximum power, the measured communication ranges remained below the minimum communication range required for reliable vehicle-guidance applications. For the mortar concrete block, the communication range was measured as ${4.80\;\times 10}^{-1}$ m in length and ${4.56\;\times 10}^{-1}$ m in width (Figure 10). The gravel concrete block achieved a slightly larger communication range of ${5.10\;\times 10}^{-1}$ m in length and ${5.45\;\times 10}^{-1}$ m in width (Figure 11). For both blocks, the origin (0, 0) of the communication range corresponds to the position of the hole of the PVC pipe opening on the surface of the concrete block. Although the measured communication ranges have not been analyzed theoretically, they represent the combined influence of the PVC pipe embedded within the concrete block, the pipe opening, and the silicone sealant used to secure the RFID tag. However, these communication ranges were insufficient for stable vehicle navigation. In the worst-case scenario, RFID antennas may fail to detect the embedded RFID tags, potentially causing the vehicle to deviate from the intended driving lane.

**Figure 12:**
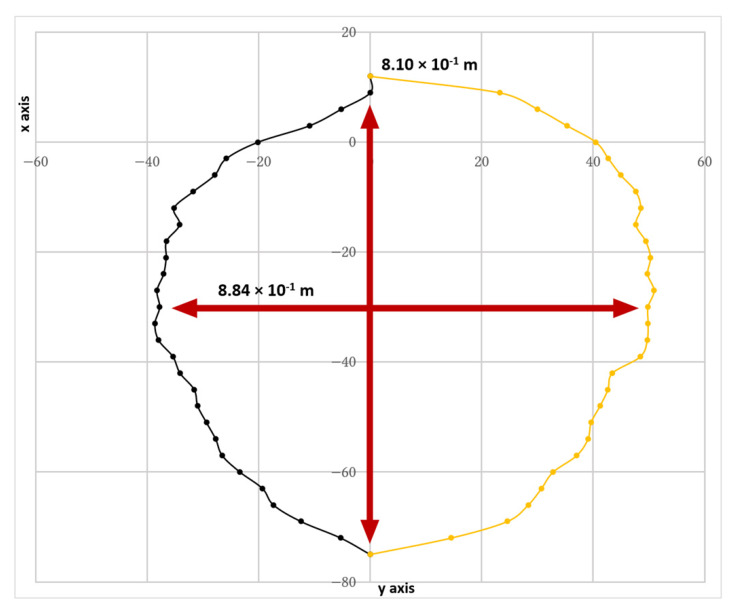
Communication range for the full-sized block.

**Figure 13:**
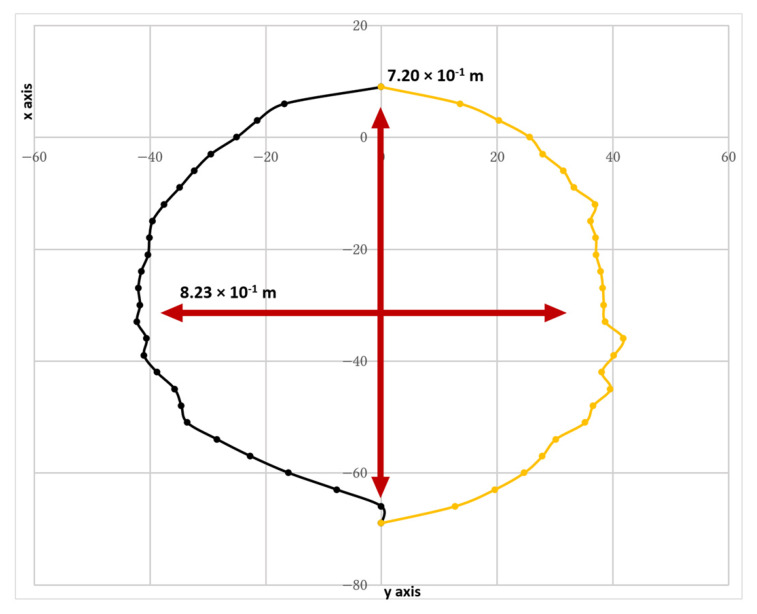
Communication range for the half-sized block.

By contrast, commercially available concrete blocks exhibited significantly larger communication ranges owing to their hollow internal structure with crushed stone No. 5 and reduced signal attenuation. As excessively large communication range may decrease the localization accuracy of the vehicle guidance system, lower antenna output powers were intentionally applied during the measurements. The full-sized commercial concrete block was evaluated at an antenna output power of 27 dBm and achieved a communication range of ${8.10\times 10}^{-1}$ m in length and ${8.84\times 10}^{-1}$ m in width (Figure 12), whereas the half-sized commercial concrete block was evaluated at an antenna output power of 24 dBm, resulting in a communication range of ${7.20\times 10}^{-1}$ m in length and ${8.23\times 10}^{-1}$ m in width (Figure 13). For both commercial blocks, the origin (0, 0) of the communication range corresponds to the position of the PVC pipe opening on the surface of the concrete block. A lower antenna output power was necessary for the half-sized commercial block because its smaller dimensions and reduced surrounding material caused less signal attenuation than that of the full-sized block. Under identical antenna power conditions, a half-sized block produces a larger communication range. Reducing the antenna output power helped constrain the communication range to values closer to the ideal target range required for precise vehicle localization while maintaining stable RFID tag detection. The experimental results for all block types are listed in Table 1. The communication range measurements were obtained under the combined influence of multiple factors, including the opening in the PVC pipe and the silicone sealant used to seal the pipe.

## 6. Geometrical Analysis of Communication Range

**Figure 14:**
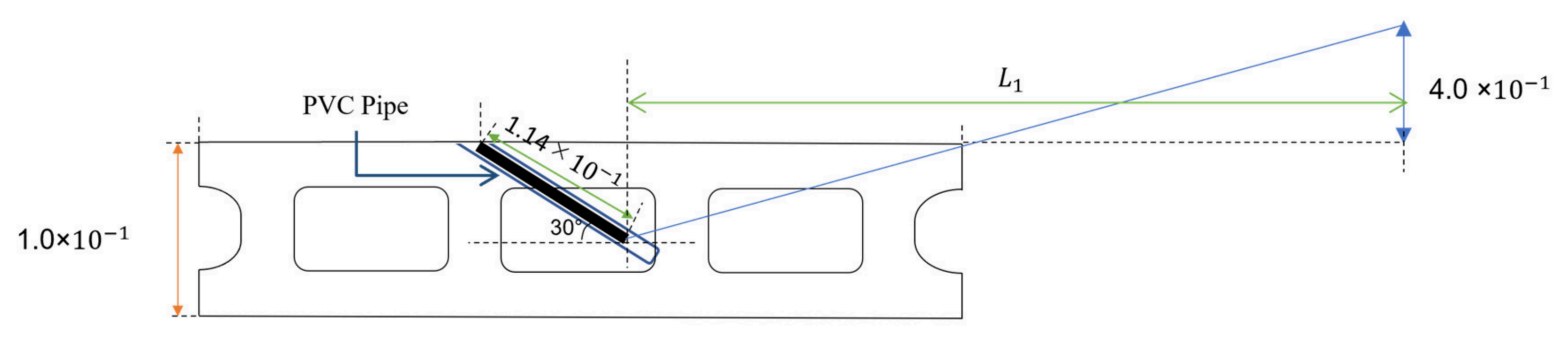
Geometric maximum communication range calculation for the full-sized concrete block.

**Figure 15:**
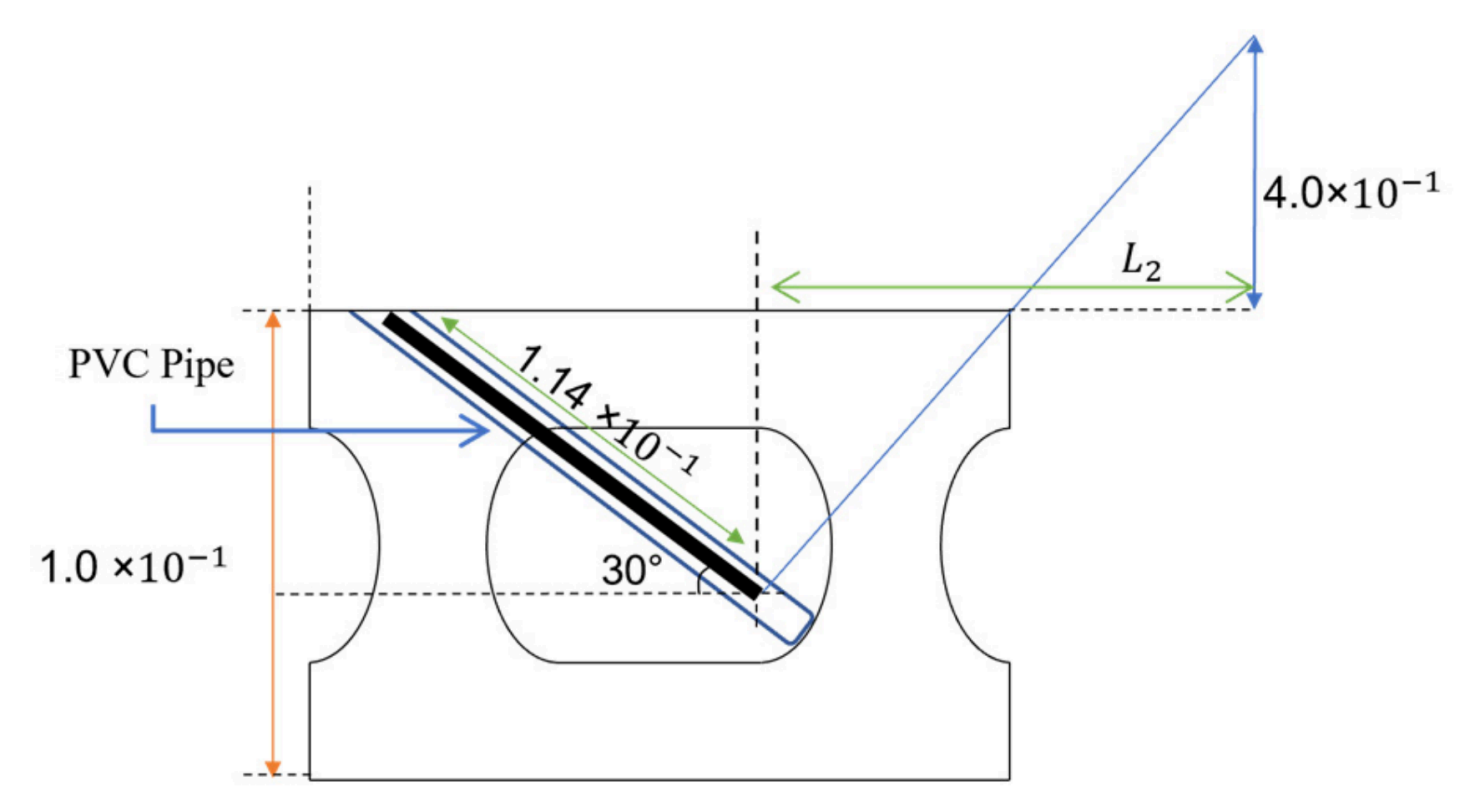
Geometric maximum communication range calculation for the half-sized concrete block.

The geometrical analysis presented in this section is intended only as a first-order feasibility estimate of the communication range. When commercially available concrete blocks are embedded in natural unpaved roads, it is important to evaluate whether the communication range is influenced by the surrounding soil conditions. In particular, the longer communication paths shown on the right side of Figure 14 and Figure 15 are of primary interest. If the communication range remains within the 0.4 m vertical region defined by the straight line connecting the bottom edge of the RFID tag to the edge of the concrete block, the electromagnetic waves transmitted between the RFID tag and the reader antenna propagate entirely through the concrete block rather than through the adjacent soil. The objective of this analysis was to determine whether the calculated range satisfies the communication requirements for accurate vehicle localization, namely, ${9.30\times 10}^{-1}$ m in the longitudinal direction and ${8.50\times 10}^{-1}$ m in the lateral direction, at an antenna height of ${4.0\times 10}^{-1}$ m above the road surface.

The model assumes that the RFID antenna is positioned at a height of 0.4 m above the ground surface and aligned parallel to the RFID tag, which is embedded at an angle of 30°.

From geometric considerations, the longitudinal communication distance for the full-sized concrete block was calculated as $L_{1}=1.472$ m, whereas that for the half-sized concrete block was $L_{2}={6.31\times 10}^{-1}$ m.

**Figure 16:**
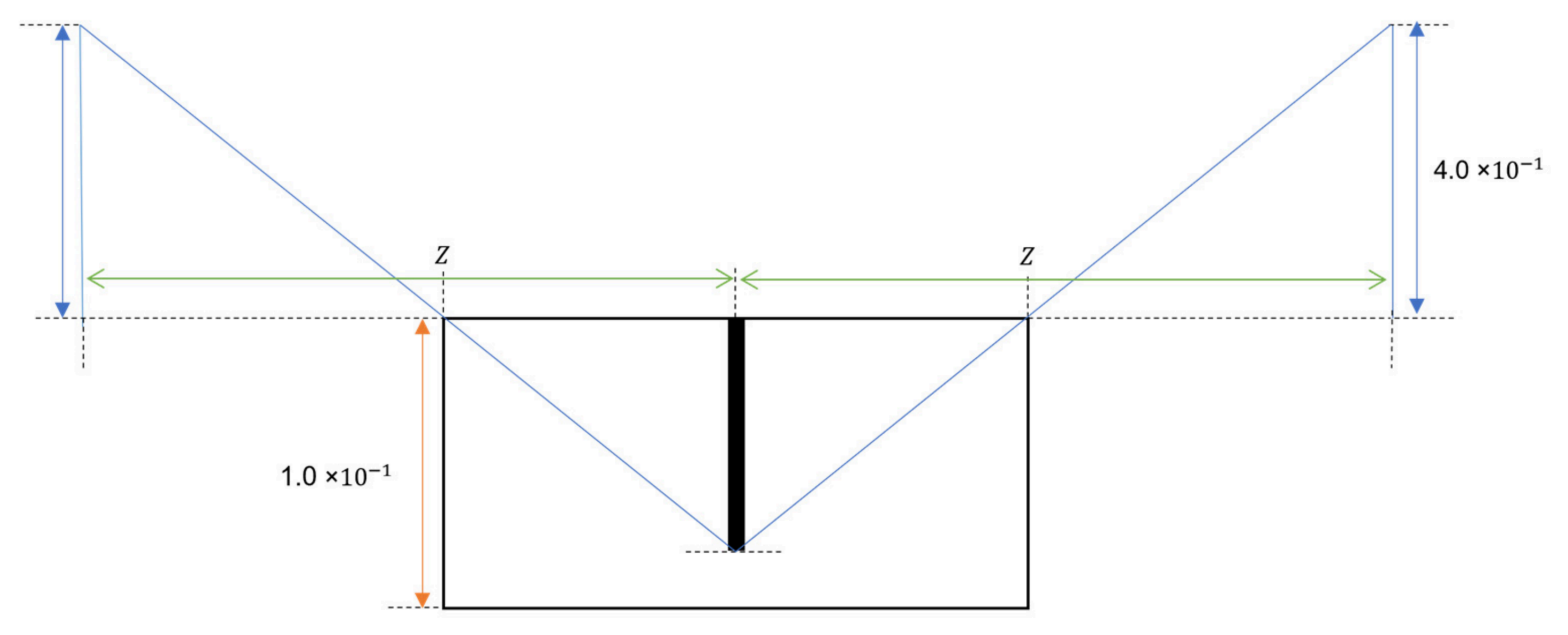
Geometric calculation of the maximum lateral communication range, identical for both half- and full-sized concrete blocks.

Similarly, in the lateral direction (Figure 16), the communication range was evaluated by considering the point located at a height of 0.4 m along the straight line connecting the bottom edge of the RFID tag to the outer edge of the concrete block. Because the full- and half-sized concrete blocks have the same width, the calculated lateral communication distance was identical for both cases, with $Z={7.61\times 10}^{-1}$ m, as shown in Figure 16.

The calculated lateral communication range was subsequently compared with the communication range required for stable vehicle navigation.

### Comparison of Geometric Calculation and Measurement of Communication Range

Based on the geometrical configurations shown in Figure 14 and Figure 15, the communication distances were calculated using trigonometric relationships. The calculated communication ranges are compared and summarized in Table 2.

Geometrical analysis confirmed that both commercial concrete block configurations provided a sufficient communication range for RFID-based vehicle navigation. The longitudinal communication range was larger for the full-sized concrete block. Although the half-sized concrete block exhibited a slightly smaller longitudinal communication range (approximately ${3.0\;\times 10}^{-2}$ m smaller), this difference does not significantly affect the RFID-based navigation performance. Even in the worst-case scenario, where the communication range was reduced by approximately ${3.0\;\times 10}^{-2}$ m in the longitudinal direction owing to environmental effects, the remaining range was still adequate for reliable vehicle guidance.

For RFID-based navigation, a sufficient communication width in the lateral direction is particularly important. The full- and half-sized concrete blocks shared the same structural configuration in the lateral direction. Therefore, the geometrical conditions provided a sufficient communication width, exceeding the measured communication ranges shown in Figure 12 and Figure 13. These results indicate that, in the lateral direction, radio waves can propagate effectively through the concrete block itself and are not significantly influenced by the surrounding natural soil.

## 7. Driving Experimental Results

**Figure 17:**
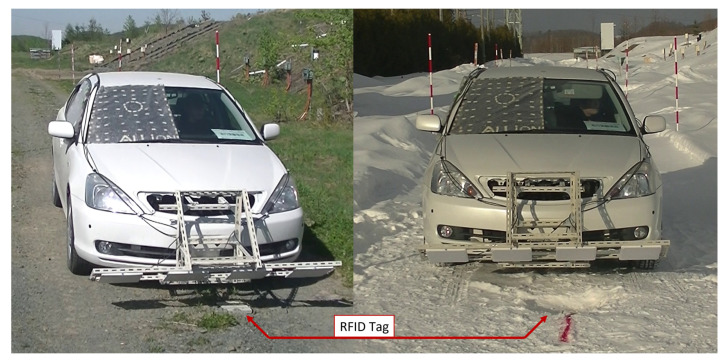
Experimental vehicle on the unpaved test lane without geocell reinforcement, showing the buried half-sized concrete block containing the embedded RFID tag.

Vehicle guidance experiments were conducted on a 50 m long natural unpaved road in where 25 blocks with RFID tags were buried at intervals of approximately 2 m (Figure 17). The driving experiments were performed during winter, spring, and summer. During the winter experiments, the road surface was covered with approximately ${1.0\times 10}^{-1}$ m of snow under an ambient temperature of approximately −4 °C. In contrast, the spring experiments were conducted under snow-free conditions at approximately 8 °C on a dry road surface under sunny weather, whereas the summer experiments were performed under cloudy conditions with light rain at that time the blocks were wet. The performance of the proposed half-sized commercial concrete block installation method was evaluated experimentally, and the results were compared with those obtained using the PVC holder method. The driving trial results are summarized in Table 3. In the half-block installation, the RFID tags buried along the driving lane were installed with positional accuracy, with a maximum deviation of approximately ${5.0\times 10}^{-2}$ m from the centerline of the lane.

**Figure 18:**
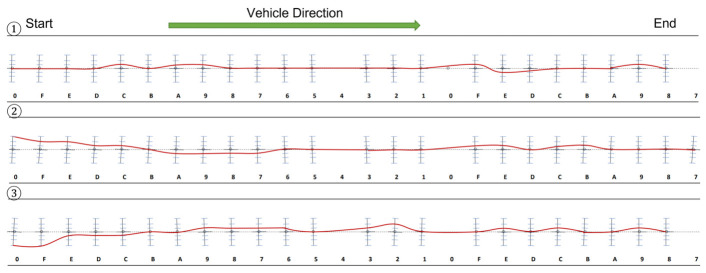
The red lines represent the vehicle trajectory with respect to the starting point from the center, left side, and right side of the lane.

The experiments were conducted by four different drivers, each performing three trials starting from the center, left, and right positions within the lane, as shown in Figure 18. During each trial, the driver gradually steered the vehicle toward the lane center by following the guidance information displayed on the graphical user interface (GUI). Although the resulting trajectories do not appear perfectly straight, this is primarily due to the small positional deviations of the RFID tags from the intended lane centerline, which arose from manual installation. Consequently, the estimated vehicle trajectory reflects these installation tolerances rather than inaccuracies in the guidance system.

Figure 18 shows the driving trajectories from three trials conducted by the same driver, with the vehicle departing from the center, left, and right positions of the start line. The trajectories were reconstructed using the positions of the reader antenna. Although slight differences are observed in the final positions of the trajectories, these variations result from the stopping locations instructed to the driver. Consequently, in some trials, the final RFID tag in the lane was not detected. These differences are therefore attributable to the experimental procedure rather than any limitation or malfunction of the vehicle navigation program or RFID tag. The relationship between the estimated and actual positions obtained using the four-antenna localization system has been reported in [11].

Table 3 shows that the half-sized concrete block installation method produced larger vehicle-tracking deviations than the PVC pipe holder method at the end of the trials. Although both methods achieved a minimum deviation of 0 m, the half-block system exhibited a higher maximum deviation of ${2.14\times 10}^{-1}$ m, a standard deviation of ${5.56\times 10}^{-2}$ m, and an average deviation of ${1.04\times 10}^{-1}$ m compared with the PVC pipe holder method. The wider 95% confidence interval of the half-block system also indicated greater variability in vehicle guidance accuracy. Furthermore, the 95% confidence intervals of the two installation methods do not overlap, suggesting that the larger average deviation observed for the half-sized block method was statistically consistent rather than being attributable to random experimental variation. This difference is attributed to slight variations in the positioning of the embedded RFID tags within the driving lane. Nevertheless, the communication range measurements presented in Table 3 demonstrated comparable RFID communication performance for both installation methods, indicating that additional improvements in installation accuracy could reduce the guidance error of the half-sized block method.

Despite the increased deviation, the measured errors remained within the allowable range required for practical vehicle-guidance applications. Although the half-sized concrete block method exhibited a larger average tracking error than the PVC holder method, the maximum deviation remained approximately 0.21 m. Although this value may be considered an outlier based on the standard deviation and confidence interval, the corresponding positioning error is sufficiently small to keep the vehicle within the driving lane. Overall, these results demonstrate that the proposed half-sized commercial concrete block installation method provides adequate vehicle-guidance performance while simplifying installation and improving structural stability by eliminating the need for a separate PVC pipe holder and geocell reinforcement.

## 8. Discussion

The results of this study demonstrate that the communication performance of buried RFID systems is strongly influenced by both the electromagnetic properties of the embedding material and the structural configuration used to stabilize the RFID tags. Dense mortar structures significantly attenuate electromagnetic waves because cementitious materials absorb and weaken the transmitted signal, reducing the communication range of the embedded passive UHF RFID tags, resulting in communication ranges that are insufficient for reliable vehicle localization. Although the inclusion of coarse aggregates in the gravel concrete block slightly improved the communication performance, the obtained communication range remained below the threshold required for stable vehicle guidance.

By contrast, commercially available concrete blocks achieved communication ranges close to the ideal communication range required for accurate vehicle positioning. A possible reason for this improvement is the internal hollow structure commonly present in commercial concrete blocks, which reduces electromagnetic attenuation compared with dense mortar materials. In addition, the standardized geometry of commercial blocks improved the consistency of RFID tag placement and communication characteristics.

The results also indicated that controlling the antenna output power is essential for maintaining the communication range required for vehicle localization. Excessively large communication range may reduce the positioning accuracy because multiple antennas can detect the same RFID tag over a wider region. Therefore, optimizing both the embedding structure and the antenna output power is necessary to achieve stable and accurate vehicle guidance.

The half-sized commercial concrete block demonstrated several practical advantages over the previously developed PVC holder method. Its compact size simplified installation and minimized interference with the geocell structure while maintaining stable RFID communication performance. Furthermore, because the concrete block provides structural protection and support for the embedded RFID tag, the proposed method has the potential to enable RFID installation on unpaved roads without requiring full geocell reinforcement. Table 4 summarizes the differences in installation strategy, application environment, and research objectives between the previous and present RFID-based vehicle navigation studies.

Since this study primarily focuses on driving on unpaved roads covered in snow during the winter under conditions of poor visibility, vehicle speeds are low.

According to the Impinj documentation [26], the RFID reader/writer is capable of interrogating RFID tags at a rate of up to 1100 reads per second. The number of successful reads for a given tag depends on the distance over which the antenna remains within the communication range and the vehicle speed. In the present system, the maximum communication range for a half-sized concrete block is approximately 0.72 m. For example, at a travel speed of 20 km/h, the antenna remains within the communication range for approximately 129.6 ms, allowing approximately 142 interrogation attempts. Even if the vehicle deviates from the intended path and only half of the communication range is traversed, approximately 71 interrogation attempts remain possible, which is considered sufficient for reliable tag detection.

One potential limitation is the antenna switching time of the Impinj reader/writer. According to the manufacturer’s specifications, switching between antenna ports requires approximately 25 ms [27]. With the four-antenna configuration used in this study, a complete switching cycle requires approximately 75 ms before returning to the first antenna. At a vehicle speed of 20 km/h, the antenna remains within the communication range for approximately 129.6 ms, providing sufficient time for all four antennas to interrogate the RFID tag during a single pass.

During the vehicle experiments, a whiteout environment was simulated by completely blocking the driver’s forward field of view. Under these conditions, the maximum operating speed was approximately 10 km/h, and no missed RFID tag detections were observed. Furthermore, RFID tags were conservatively installed at 2 m intervals; therefore, even if a single tag is missed, the following tag is likely to be successfully detected. For applications requiring operation at higher vehicle speeds, the antenna output power can be increased to extend the communication range, and the output power can be adjusted directly within the driver’s program. In such applications, the previously developed configuration consisting of two antennas and three RFID tags installed at 2 m intervals may also be adopted to provide additional communication redundancy.

Although the proposed installation method demonstrated stable communication performance during vehicle experiments conducted in light rain, where the concrete blocks became wet without affecting RFID detection, standing water surrounding the embedded concrete blocks represents a potential limitation. Water has a high dielectric constant and loss factor at UHF frequencies, which can significantly attenuate electromagnetic waves and reduce the communication reliability of passive RFID tags. To mitigate this issue, future implementations will incorporate waterproof concrete block assemblies using waterproof sheets and water-resistant surface coatings to minimize water ingress and preserve stable communication performance in an environment surrounded by water conditions. Table 4 summarizes our research gaps and contributions.

## 9. Conclusions

This study investigated several RFID tag installation methods for vehicle navigation on unpaved roads, focusing on maintaining the communication range required for stable vehicle localization while simplifying the installation procedures.

The experimental results indicated that the mortar concrete blocks caused strong electromagnetic attenuation and produced communication ranges significantly smaller than the ideal range required for vehicle guidance. The gravel concrete blocks slightly improved the communication performance because of the additional internal voids introduced by the coarse aggregates; however, the communication range remained insufficient for reliable operation.

Commercially available concrete blocks achieved communication ranges close to the ideal communication range required for accurate vehicle localization. Furthermore, the experimentally measured communication ranges closely matched the geometrically predicted results, confirming the validity of the proposed geometrical analysis method.

Among the evaluated configurations, the half-sized commercial concrete block provided the best balance between communication performance and installation practicality. The smaller block simplified installation while maintaining stable RFID communication characteristics. Vehicle guidance experiments conducted under snowy, unpaved road conditions confirmed successful navigation without lane departure.

The results demonstrated that commercially available concrete blocks, particularly the half-sized configuration, provide a practical and durable solution for RFID-based vehicle navigation on unpaved roads. The proposed method also has potential applicability to unpaved roads without geocell reinforcement, which may simplify the RFID infrastructure deployment on temporary roads, forest roads, and unpaved natural soils.

Future studies will focus on evaluating the long-term durability, communication stability under environmental deformation, and performance under varying snow and moisture conditions.

## Figures and Tables

**Table 1:** Measured communication ranges and antenna output power for different RFID tag embedding options.

Block Type	Width [m]	Length [m]	Antenna Output Power (dBm)
Mortar concrete	${4.56\times 10}^{-1}$	${4.80\times 10}^{-1}$	30
Gravel concrete	${5.45\times 10}^{-1}$	${5.10\times 10}^{-1}$	30
Commercial concrete (Full-sized)	${8.84\times 10}^{-1}$	${8.10\times 10}^{-1}$	27
Commercial concrete (Half-sized)	${8.23\times 10}^{-1}$	${7.20\times 10}^{-1}$	24

**Table 2:** Geometrically calculated communication ranges.

	Longitudinal [m]	Lateral [m]
Full-sized	1.472	${7.61\times 10}^{-1}$
Half-sized	${6.31\times 10}^{-1}$	${7.61\times 10}^{-1}$

**Table 3:** Comparison of the results of driving experiments using half-sized concrete blocks versus using PVC pipe holders.

	Half-Sized Block	PVC Pipe
Total trials	101	102
Minimum deviation [m]	0	0
Maximum deviation [m]	${2.14\times 10}^{-1}$	${1.50\times 10}^{-1}$
Standard deviation [m]	${5.56\times 10}^{-2}$	${3.73\times 10}^{-2}$
Average deviation [m]	${1.04\times 10}^{-1}$	${4.46\times 10}^{-2}$
Confidence Interval (95%) [m]	$[{9.29\;\times 10}^{-2}$$,\;{1.14\;\times 10}^{-2}$]	$[{3.73\;\times 10}^{-2}$$,\;{5.18\;\times 10}^{-2}$]

**Table 4:** Comparison of previous RFID vehicle navigation studies and the proposed method.

Reference	Application	Road Environment	RFID	Installation Method	Vehicle Guidance Validation	Comments
[5]	Vehicle navigation	Asphalt	Passive UHF	30° buried	Yes	2 antennas
[10]	Communication optimization	Asphalt	Passive UHF	30°, 60°, 90° buried	No	Communication only
[11]	Vehicle navigation	Asphalt	Passive UHF	30° buried	Yes	4 antennas
[12]	Vehicle navigation	Unpaved With Geocell	Passive UHF	PVC holder + geocell	Yes	Installation complexity
This study	Vehicle navigation	Unpaved natural soil	Passive UHF	Commercial concrete block	Yes	Simplified installation

## Data Availability

All data and materials related to this study are available upon request.

## Abbreviations

The following abbreviations were used in this manuscript:

LiDAR	Light detection and ranging
GNSS	Global navigation satellite system
UHF	Ultrahigh frequency
RFID	Radio-frequency identification
GPS	Global positioning system
RSSI	Signal strength indicators
RF	Radio frequency
SLAM	Simultaneous localization and mapping
PVC	Polyvinyl chloride
INS	Inertial navigation system
